# Autoimmune Hepatitis Refractory to Treatment Due to Underlying Grave’s Disease

**DOI:** 10.7759/cureus.4598

**Published:** 2019-05-04

**Authors:** Rahul Sawhney, Sadhna Dhingra, Gagan K Sood

**Affiliations:** 1 Internal Medicine, Baylor University Medical Center, Dallas, USA; 2 Anatomic Pathology, Baylor College of Medicine, Houston, USA; 3 Abdominal Transplantation, Baylor College of Medicine, Houston, USA

**Keywords:** hepatitis, autoimmune hepatitis, liver disease, grave’s disease, hyperthyroidism, mmi, aih, propylthiouracil, methimazole, ptu

## Abstract

A 24-year-old Hispanic woman presented to our facility with a two-week history of abdominal pain, nausea, vomiting, diarrhea, jaundice, and scleral icterus. Initial laboratory workup revealed elevated transaminases, direct hyperbilirubinemia, and positive anti-smooth muscle antibody. Liver biopsy confirmed the diagnosis of autoimmune hepatitis and our patient was started on oral prednisone therapy. Her liver enzymes initially began to normalize but then spontaneously started up-trending. She was subsequently readmitted to the hospital for further management, at which time she also complained of palpitations, heat intolerance, and sweating. Laboratory workup revealed hyperthyroidism secondary to Grave’s disease. Our patient was not a candidate for methimazole or propylthiouracil treatment due to her hepatic dysfunction, so she was started on hydrocortisone due to its secondary effect of decreased conversion of thyroxine to triiodothyronine. She achieved biochemical remission of her autoimmune hepatitis on this regimen and was transitioned back to oral prednisone therapy. Her liver enzymes normalized once she underwent radioactive iodine ablation of her thyroid. This clinical course suggests that autoimmune hepatitis with concurrent Grave’s disease may be refractory to treatment until the underlying hyperthyroid state is corrected.

## Introduction

Autoimmune hepatitis (AIH) is a chronic inflammatory condition characterized by hepatocellular inflammation secondary to autoantibody-induced lymphocytic proliferation [1]. The presentation of AIH is varied, ranging from patients who are asymptomatic to those who present with acute liver failure [2]. AIH is commonly known to be associated with other autoimmune diseases [3]. We present a case of AIH with associated Grave’s disease in which the underlying hyperthyroidism seemingly contributed to progressive liver dysfunction.

## Case presentation

A 24-year-old Hispanic woman with a past medical history of post-partum pre-eclampsia presented to the emergency department with a two-week history of abdominal pain, nausea, vomiting, diarrhea, jaundice, and scleral icterus. On a review of systems, she denied recent travel, sick contacts, hepatotoxic medications or supplements, or parenteral exposure risks. She reported a distant history of tattoos placed with clean needles and had undergone a cesarean section three months prior, with the delivery of a healthy baby boy. Aside from obesity, she denied other risk factors for liver disease.

Her physical exam was unremarkable except for obesity, jaundice, and scleral icterus. The patient had no exophthalmos, goiter, or tachycardia. Initial laboratory workup revealed elevated liver function tests, including aspartate transaminase (AST) 1,041 IU/L (8-48 IU/L), alanine transaminase (ALT) 809 IU/L (7-55 IU/L), total bilirubin 11.5 mg/dL (0.1-1.2 mg/dL), direct bilirubin 8.7 mg/dL (<0.3 mg/dL), and prothrombin time 15.1 seconds (9.5-13.8 seconds). Anti-smooth muscle antibody (ASMA) was positive and immunoglobulin G (IgG) was elevated at 2,288 mg/dL (767-1,590 mg/dL). Anti-nuclear/anti-mitochondrial antibodies and hepatitis viral serologies were negative. Iron/copper studies were within normal limits. Thyroid studies were not performed at this time, as she did not exhibit any clinical evidence of thyroid dysfunction.

**Figure 1:**
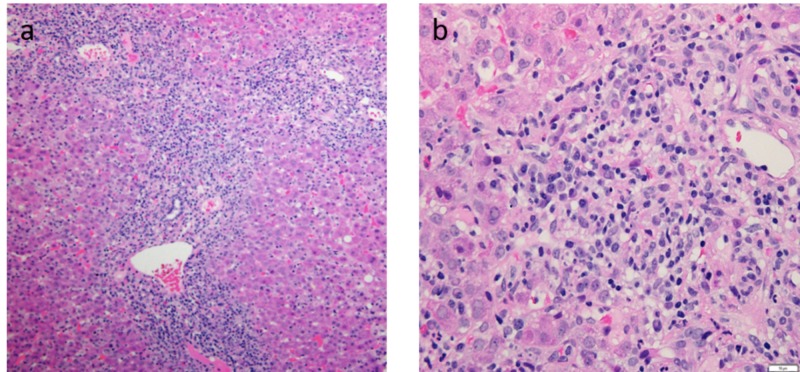
Liver biopsy slides of findings consistent with autoimmune hepatitis. (a) Portal expansion by chronic lymphoplasmacytic inflammation with moderate to severe interface hepatitis. (b) Inflammatory infiltrate includes clusters of plasma cells admixed with lymphocytes and few eosinophils.

Subsequently, an ultrasound-guided core needle liver biopsy was performed, which showed interface hepatitis with extensive portal lymphocytic infiltration, mild to moderate (Grade 2-3) periportal and lobular inflammation, portal fibrosis, and early (Stage 1-2) portal-to-portal septa (Figure 1), consistent with a diagnosis of autoimmune hepatitis.

The probability of our patient having AIH was calculated using a standardized scoring system. Based on the presence of ASMA, IgG elevation >1.1 times the upper limit of normal, histology compatible with a diagnosis of AIH, and negative viral serologies, the patient’s score was 6 as per the simplified AIH scoring system. This was indicative of a probable diagnosis of AIH [2,4-5]. Human leukocyte antigen (HLA) allele was not tested.

**Figure 2:**
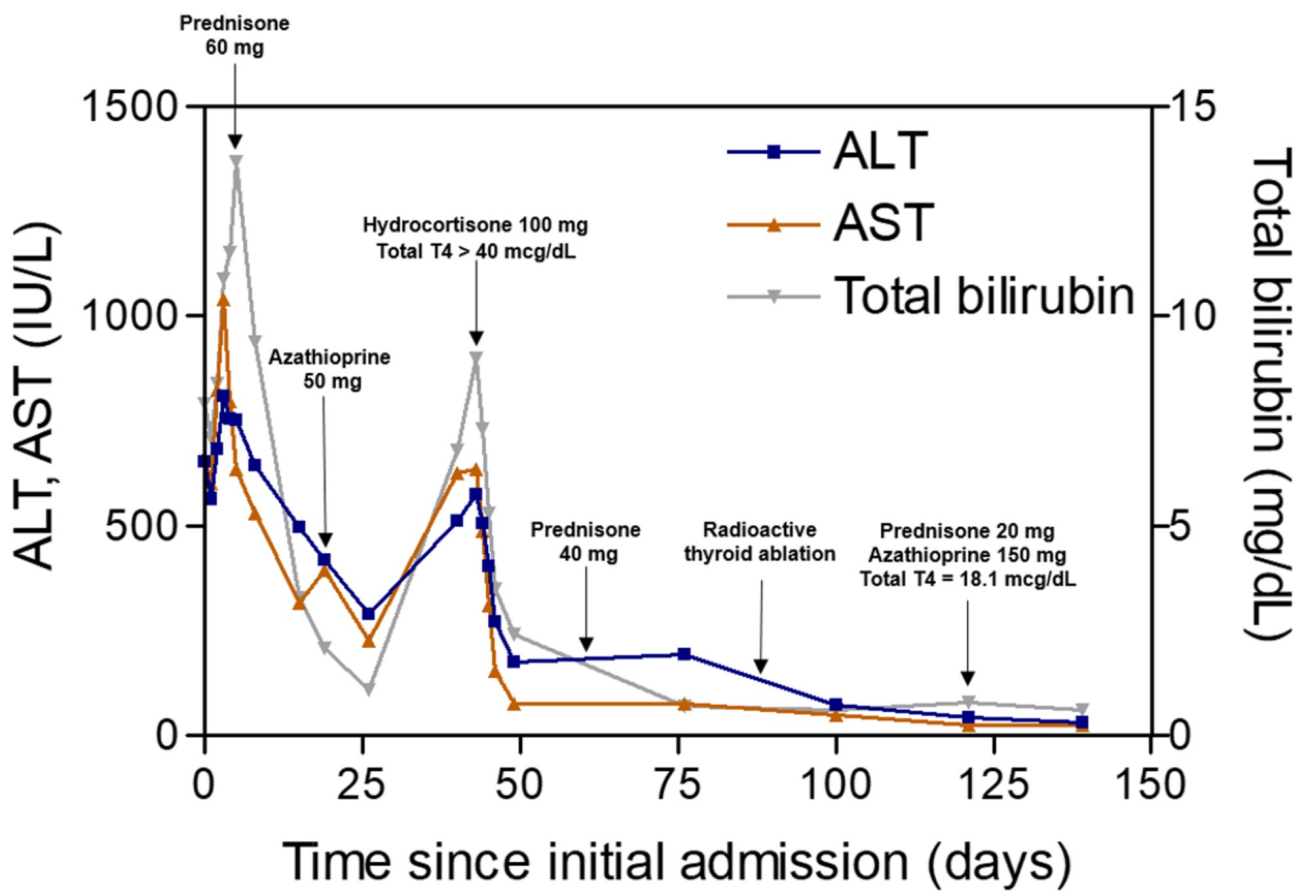
Patient’s clinical course since time of admission. ALT: alanine aminotransferase; AST: aspartate aminotransferase; T4: thyroxine.

Treatment was started with high-dose prednisone to be tapered from 60 mg to 40 mg after two weeks. Azathioprine 50 mg daily was added after three weeks. Initially, her transaminases and total bilirubin trended downward, but these values sharply increased at the six-week mark (Figure 2). She also complained of palpitations, sweating, and heat intolerance, which prompted readmission for further evaluation. Additional workup revealed underlying hyperthyroidism with thyroid-stimulating hormone (TSH) <0.01 µIU/mL (0.4-5.5 µIU/mL), freetriiodothyronine (T3) 11.5 pg/mL (2.8-4.4 pg/mL), and free thyroxine (T4) 39.5 ng/dL (0.9-1.7 ng/dL). Grave's disease was confirmed with a radioactive iodine uptake of 44% (8-25%) and positive thyroid-stimulating immunoglobulin.

Given her hepatic function, the patient was not a candidate for standard therapy with methimazole or propylthiouracil. She was instead started on high-dose hydrocortisone, which provided an added benefit of decreased peripheral conversion of T4 to T3 [6]. Her hyperthyroid symptoms improved while AST, ALT, and total bilirubin decreased by nearly 75% within one week. This represented the first time in three months that the patient’s transaminases dropped below 200 IU/L. Subsequently, she underwent radioactive thyroid ablation and began levothyroxine supplementation. Her AST, ALT, and total bilirubin also normalized - signifying the biochemical remission of AIH - once she received the ablation. Afterward, the patient remained stable on standard immunosuppressive therapy, with 20 mg prednisone and 150 mg azathioprine daily.

## Discussion

Our patient was diagnosed with autoimmune hepatitis based on positive autoimmune serology (anti-smooth muscle antibody), elevated IgG, and compatible histopathological findings. Standard immunosuppressive therapy with prednisone and azathioprine yielded a suboptimal response. An additional diagnosis of Grave’s disease was made based on clinical findings, low TSH, elevated free T3 and T4, positive thyroid stimulating immunoglobulin, and increased radioactive iodine uptake.

Concomitant autoimmune disorders, including thyroid disease, diabetes, inflammatory bowel disease, and rheumatoid arthritis, have been described in nearly 40% of patients with AIH. However, the concurrence of additional autoimmune diseases does not usually alter the clinical course or severity of AIH [3,7].

Initial treatment for AIH includes high-dose prednisone daily, to which our patient had a suboptimal response [2]. Upon her diagnosis of Grave’s disease, our patient was started on intravenous hydrocortisone, as methimazole and propylthiouracil were contraindicated given her liver function. This regimen led to markedly improved control of the patient’s AIH. Hydrocortisone, along with suppressing the immune system, prevents the conversion of T4 to T3 and can ameliorate a hyperthyroid state [6].

Hyperthyroidism has been reported to be associated with hepatic function abnormalities through several mechanisms of liver dysfunction. These include liver abnormalities due to hyperthyroidism alone, liver damage related to heart failure and hyperthyroidism, and concomitant liver disease in the setting of hyperthyroidism [2]. Our patient had no signs of heart failure and the persistent elevation of her liver aminotransferases despite immunosuppressive therapy was attributed to the associated hyperthyroid state.

Biochemical remission was achieved with adequate control of the underlying hyperthyroidism and maintained with low-dose prednisone and azathioprine. Our patient achieved a euthyroid state after radioactive ablation of her thyroid gland and subsequent oral levothyroxine supplementation.

## Conclusions

Our case highlights that an underlying hyperthyroid state can contribute to hepatic dysfunction and may cause suboptimal responsivity of AIH to standard immunosuppressive therapy. The recognition and management of associated hyperthyroidism can aid in achieving an optimal biochemical response and avoiding unnecessary incremental increases in the immunosuppressive regimen. Based on our findings, we recommend that patients with AIH refractory to therapy be evaluated for possible thyroid disease.
